# Characterization of Novel Angiotensin-Converting Enzyme Inhibitory Peptides

**DOI:** 10.1021/acsmedchemlett.5c00569

**Published:** 2025-11-26

**Authors:** Camila Innocente-Alves, Sara Luísa Sulzbach, Emerson Gonçalves Moreira, Raul Izidoro Carneiro, Lucélia Santi, Hugo Verli, Walter Orlando Beys-da-Silva

**Affiliations:** † Faculdade de Farmácia, 28124 Universidade Federal do Rio Grande do Sul, Porto Alegre 90010-150, Brazil; ‡ Programa de Pós-Graduação em Biologia Celular e Molecular, 28124 Universidade Federal do Rio Grande do Sul, Porto Alegre 90010-150, Brazil; § Centro de Biotecnologia (CBiot), 28124 Universidade Federal do Rio Grande do Sul, Porto Alegre 90010-150, Brazil

**Keywords:** Angiotensin-Converting Enzyme, Hypertension, Bioactive Peptides, Antihypertensive, Molecular Docking

## Abstract

Hypertension is implicated in the highest number of deaths worldwide. Despite awareness of its complications and the availability of several antihypertensive treatments, hypertension remains poorly controlled, often due to adverse effects that can hinder adherence. Angiotensin-converting enzyme (ACE), a key enzyme of the renin-angiotensin system (RAS), is an important therapeutic target. Bioactive peptides have been extensively researched for their biological activities, including their antihypertensive potential. Here, we describe two novel peptides, MSFLEHFLELK (PepDB_AHP1) and VWTNCYHLYPAH (PepDB_AHP4). Both peptides interact with residues at ACE’s active site, such as His353, Ala354, and Val380. IC_50_ values were 331.2 and 88.63 μM, respectively. These peptides may serve as models for further optimization aimed at the development of novel ACE-inhibitory drugs.

## Graphical abstract

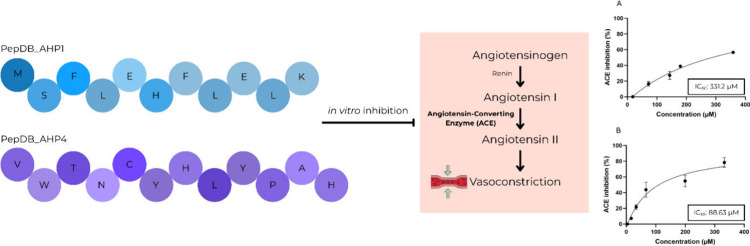

Hypertension, commonly termed “high blood pressure”, is defined as a systolic blood pressure equal or greater than 140 mmHg and a diastolic blood pressure equal or greater than 90 mmHg. Although almost half of adults living with hypertension are unaware of their condition,^1^ it is the major cause of premature death worldwide. Accordingly, hypertension management is also responsible for massive expenses. For instance, in 2018 in Brazil, a country with high incidence of hypertensive patients, the public health system (Sistema Único de Saúde–SUS) spent BRL$ 3.45 billion to manage hypertension, diabetes, and obesity, of which 59% corresponded to hypertension management, that is, BRL$ 2 billion.^2^

Among the many antihypertensive drug classes available nowadays, angiotensin-converting enzyme inhibitors (ACEis) are one of the most prescribed drugs.^3^ This class of drugs targets the renin-angiotensin system (RAS), which plays an important role in blood pressure regulation. When there is a decrease in blood pressure, the kidney releases renin, which will convert angiotensinogen (AGT) into angiotensin I (Ang I). Then, angiotensin-converting enzyme (ACE) will convert Ang I into angiotensin II (Ang II), a potent vasoconstrictor.^4^ Besides converting Ang I into Ang II, ACE also degrades vasodilator peptides, such as bradykinin in the kallikrein–kinin system.^5^ Therefore, this enzyme is considered a protagonist in hypertension management.

Despite the wide range of antihypertensive drugs available, hypertension is poorly controlled. In fact, only one in five hypertensive adults have the condition under control.^1^ Also, adverse effects may lead to treatment abandonment and therefore contribute to hypertension worsening. Regarding ACE inhibitors, dry cough is the most reported adverse effect,^6^ which occurs due to bradykinin accumulation in nonselective drugs. As commercial ACE inhibitors are peptide-derived molecules, several groups have researched bioactive peptides with ACE-inhibitory activity. These peptides are obtained from several sources, including animal origin such as porcine liver and placenta^7^ and *Tenebrio molitor* ^8^ and plant origin such as *Moringa oleifera* leafs^9^ and Wuyi rock tea.^10^ Recently, studies on the bioactive potential of the marine environment have also increased, represented by ACE-inhibitory peptides derived from sources such as *Nannochloropsis oculate*,^11^ swim bladders of monkfish *Lophius litulon* ^12^ and skipjack tuna *Katsuwonus pelamis*.^13^ Nevertheless, these methodologies require substantial experimental effort and financial resources, as they rely on preparation and characterization of extracts and subsequent isolation of the peptides.

In this sense, virtual screening is a helpful tool that may increase the rate of success in the research for bioactive molecules. While in vitro discovery of bioactive peptides offers significant potential, in silico approaches may optimize and accelerate this process. In fact, virtual screening has already been successfully applied to predict ACE-inhibitory peptides from garlic^14^ and *Phascolosoma esculenta*.^15^ Thus, the present study presents the development of two novel ACE-inhibitory peptides based on in silico screening of an *in-house* peptide database, further validated in vitro.

Molecular docking is an important in silico approach that has the ability to discriminate active compounds from decoys, if correctly performed,^16^ and therefore increases the rate of success of a potential therapeutic compound. Thus, we applied molecular docking in a previous virtual screening experiment on an *in-house* library,^17^ from which the peptides PepDB_AHP1 and PepDB_AHP4 were selected. Interactions between the two complexes in the docking produced complexes are shown in Figure 1 and Table 1. Regarding PepDB_AHP1, its score was −9.4 kcal/mol, performing major interactions through Leu4-Val518, Glu5-His387, His6-Tyr523, Phe7-Tyr523, Leu8-Val380, Leu8-Ala354, Glu9-His353, and Leu10-Glu162 (peptide residue–enzyme residue). Regarding PepDB_AHP4, its score was −9.1 kcal/mol, presenting as major interactions residues Asn4-Glu411, Asn4-His353, Asn4-His353, Asn4-Ala354, Cys5-Tyr523, Tyr6-Ala354, Leu8-Val380, and Tyr9-Val379 (peptide residue–enzyme residue)

**Figure 1:**
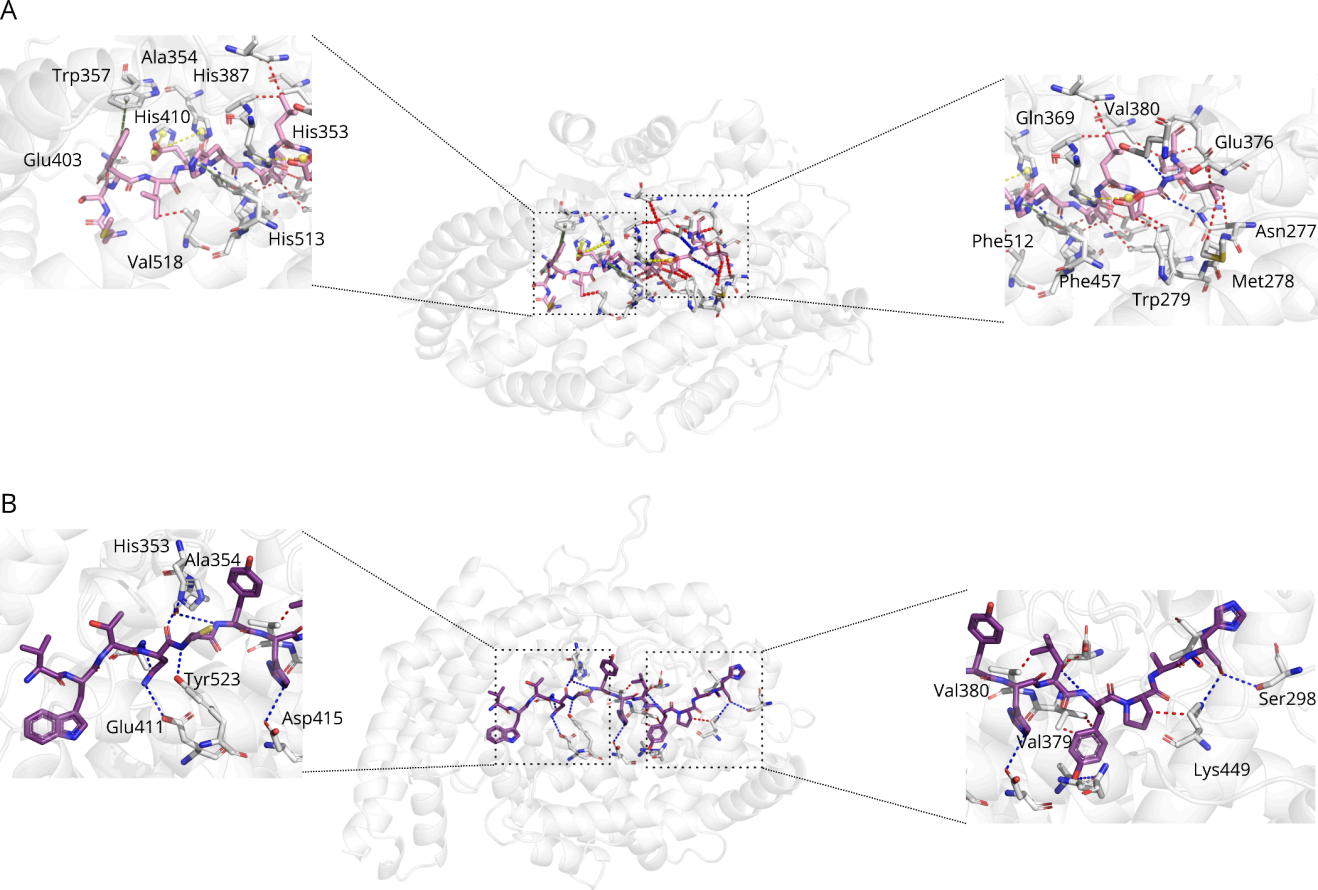
Evaluation of the molecular interactions between PepDB_AHP1 and PepDB_AHP4 and ACE. Molecular docking studies of ACE and (A) PepDB_AHP1 and (B) PepDB_AHP4. Blue dots represent hydrogen bonds, red dots represent hydrophobic interactions, yellow dots represent salt bridges, and green dots represent π-stacking interactions.

**Table 1:** Interactions between the Peptide and Enzyme Residues, According to Molecular Docking Studies

peptide	residue	interaction type	enzyme residue
PepDB_AHP1	Met1	no interaction	no interaction
	Ser2	hydrogen bond	Glu403
	Phe3	π stacking	Trp357
	Leu4	hydrophobic interaction	Val518
	Glu5	salt bridges	His387, His410
	His6	hydrogen bond, π stacking	Tyr523, Phe512
	Phe7	hydrophobic interaction	Tyr523, Phe527, Phe457
	Leu8	hydrophobic interaction	Val380, Ala354, Gln369
	Glu9	π stacking, hydrophobic interaction, hydrogen bond	His353, Trp279, Asn277
	Leu10	hydrogen bond, hydrophobic interaction	Glu 162, Thr166, Met278
	Lys11	hydrogen bond, hydrophobic interaction	Asp453, Val380, Glu376
			
PepDB_AHP4	Val1	no interaction	no interaction
	Trp2	no interaction	no interaction
	Thr3	no interaction	no interaction
	Asn4	hydrogen bond	Glu411, His353, Ala354
	Cys5	hydrogen bond	Tyr523
	Tyr6	hydrogen bond	Ala354
	His7	hydrogen bond	Asp415
	Leu8	hydrophobic interaction	Val380, Glu376
	Tyr9	hydrogen bond, hydrophobic interaction	Glu376, Ala418, Lys454, Val379
	Pro10	hydrophobic interaction	Lys449
	Ala11	no interaction	no interaction
	His12	hydrogen bond	Ser298, Lys449, Thr301

The ability of the peptides to inhibit ACE’s activity was verified, and each IC_50_ value was determined. Inhibition curves of each peptide are presented in Figure 2. PepDB_AHP1 presented the highest IC_50_ value of 461.7 μg/mL (331.2 μM), while the PepDB_AHP4 IC_50_ was 133.3 μg/mL (88.63 μM).

**Figure 2:**
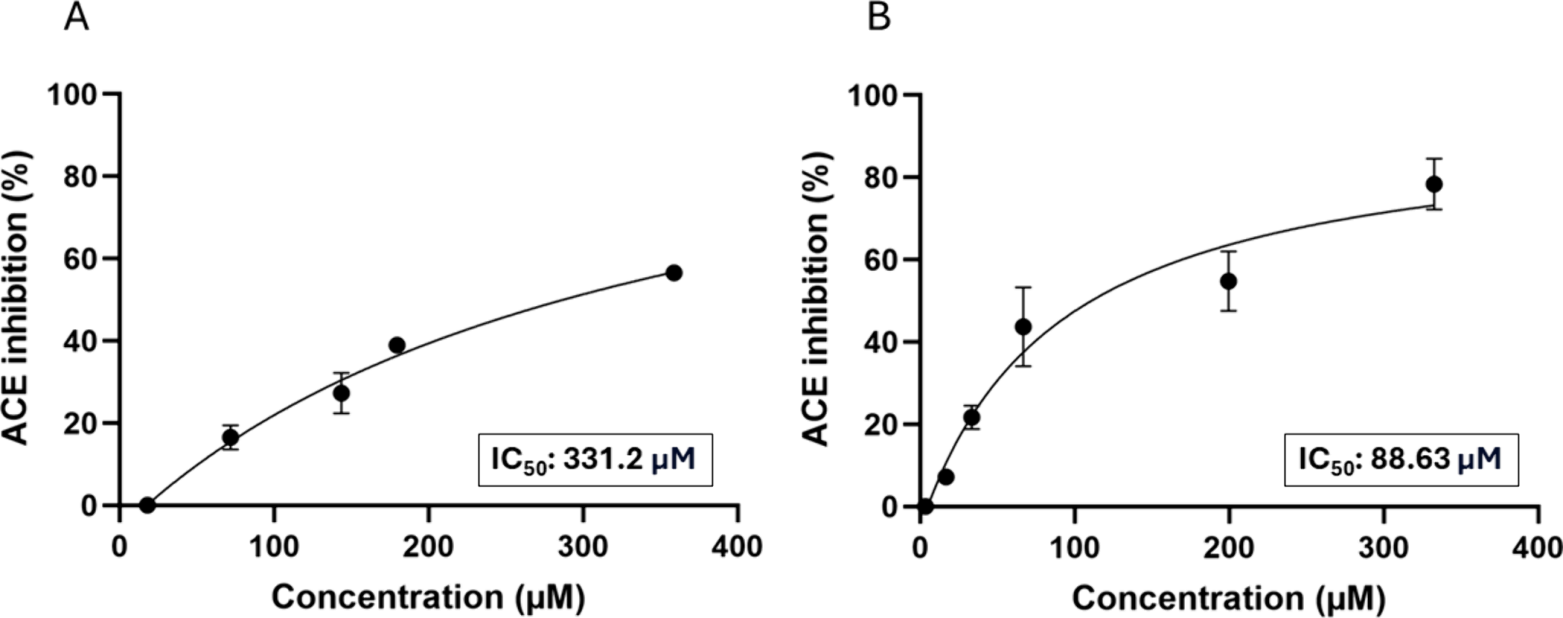
Evaluation of the peptides’ ability to inhibit ACE activity. Inhibition curves of (A) PepDB_AHP1 and (B) PepDB_AHP4.

The search for new antihypertensive molecules is intrinsically associated with the level of adverse effects shown by current antihypertensive drugs, leading to treatment abandonment. To therapeutically circumvent such side effects could improve the current status of hypertension, which is poorly controlled worldwide. Dry cough, which is the main adverse effect related to ACEis treatment, occurs due to bradykinin accumulation, as ACE is inhibited and therefore can not degrade this vasodilator peptide. Besides vasodilator activity, bradykinin is also known to induce bronchoconstriction and to participate as inflammatory mediator.^18^ Such effects originate from the fact that ACE presents two independent catalytic active domains,^19^ known as the N- and C-domains, each one presenting three binding pockets, namely S1, S1′, and S2′.^20^ ACE also presents two isoforms: testis-ACE (t-ACE) and somatic-ACE (s-ACE), where t-ACE corresponds only to the C-domain, while s-ACE presents both domains. However, the C-domain is mainly involved in Ang II formation^21^ and thus involved in blood pressure regulation. Therefore, it is reasonable to hypothesize that C-domain selective drugs would decrease dry cough as the N-domain would still be able to degrade bradykinin.

Lisinopril, a commercial ACEI, presents higher affinity to the C-domain, whereas Captopril presents higher affinity to the N-domain.^21^ Both drugs are tripeptides-analogues and present a proline-like C-terminal extremity to interact with the S2′ pocket of ACE, which is composed of Gln281, Tyr520, Lys511, His513, and His353.^22^ However, some differences should be highlighted. Captopril presents a smaller structure with a sulfhydryl group at the N-terminal to interact with the zinc ion. This molecule does not make any interactions with S1 and S1′ pockets of the enzyme, not showing selectivity among ACE domains. In turn, Lisinopril presents a larger structure but with no available group to interact with the zinc ion. While both Lisinopril and Enalaprilat interact with the S1 pocket, represented by Ala354, Glu384, and Tyr523, Enalaprilat does not interact with the S1′, while Lisinopril does, represented by Glu162. Lisinopril presents a lysine-like structure to interact with the S1′ pocket due to ionic interactions between Asp377 and the lysyl nitrogen.^20^ It is believed that the voluminous group at the portion of the peptide that interacts with the S1′ provides a higher selectivity for the C-domain, as the S1′ of the N-domain cannot accommodate bulky groups due to the exchange of Asp377 for a glutamine, which occludes the S1′.^20^ Other C-domain exclusive residues include Phe391, Val379, Val380,^23^ Val518 and Glu376.^24^ Interestingly, PepDB_AHP1 interacts with Val380, Val518, and Glu376, while PepDB_AHP4 interacts with Glu376 and both Val379 and Val380. As HHL, the substrate used in this study, is a C-domain selective substrate,^25^ in addition to the interactions with these specific residues, could raise the hypothesis of a possible C-domain selectivity. Also, the molecular docking performed in our study used the testicular ACE crystal (PDB entry: 1UZE), which corresponds to the C-domain sequence and supports this hypothesis.

Aiming to develop C-domain selective compounds, structural aspects from the ACE-Lisinopril complex should be taken into consideration in the design of screening of molecules. Also, it has already been described that peptides presenting short sequences, hydrophobic and positively charged amino acids at the C-terminal present high affinity to the enzyme, since the three residues in the C-terminal play an important role in competitive binding to the active site of the enzyme.^26^ When analyzing the peptides described in this study, both peptides present short sequences (11–12 amino acids), with positively charged amino acids at the C-terminal (lysine and histidine). Both peptides present hydrophobic profiles, as PepDB_AHP1 presents two phenylalanines, one methionine, and three leucines, for example, while PepDB_AHP4 presents one tryptophan, two tyrosines, and one leucine in its sequences. On the other hand, dicarboxylic amino acids at the C-terminal are less favorable for the inhibitory activity.^26^ As PepDB_AHP1 presents a glutamic acid at the C-terminus, it could explain its higher IC_50_ value when compared to PepDB_AHP4, since the Glu residue interacts with only one active site residue, His353.

PepDB_AHP1 interacts with His353 via hydrogen bond and π-stacking in the enzyme S2 pocket, in addition to hydrogen bonds with Tyr523 and hydrophobic interactions with Ala354 at the S1 pocket and hydrogen bond with Glu162 at the S1′ pocket. Additionally, His387 coordinates the zinc ion, and the peptide interacts with this residue via salt bridges. As for PepDB_AHP4, in addition to hydrogen bonds with Tyr523, Ala354 and His353 at the active site of the enzyme, it also interacts with Glu411 via a hydrogen bond, which coordinates the zinc ion. The peptides also share interactions with Val379 and Val380 via hydrophobic interactions. Of the 22 interactions performed by PepDB_AHP1, only 5 of them are hydrogen bonds. As for PepDB_AHP4, hydrogen bonds are predominant interactions; of the 16 interactions performed by this peptide, 12 are hydrogen bonds. Although hydrophobic interactions were for a long time described as the major forces involved in protein stability, hydrogen bonds are now known as comparable forces.^27,28^ Hydrogen bonds have also been described as important factors in stabilizing the interaction between ACE and inhibitory peptides. Two patatin peptides, WG and PRY, performed four and six hydrogen bond interactions with ACE. In agreement, PRY presented higher inhibitory potency, represented by its lower IC_50_ value.^29^ Two peptides derived from peony seed, VLSGF and HWS, were also in agreement, with 6 and 3 hydrogen bonds with ACE, respectively, in agreement with VLSGF lower IC_50_.^30^ Therefore, since PepDB_AHP4 performs more hydrogen bonds than PepDB_AHP1, the IC_50_ of PepDB_AHP4, which is lower than that of PepDB_AHP1.

Many ACE-inhibitory peptides are described in the literature, with IC_50_ values ranging from millimolar to micromolar. Four peptides identified in black tea presented IC_50_ values of 210.0 ± 18.3, 178.9 ± 5.2, 196.3 ± 2.9, and 121.1 ± 3.4 μM.^31^ Interestingly, these peptides did not interact with the active site, thus corresponding to allosteric inhibitors in vitro. ACE-inhibitory peptides obtained from walnut protein isolate presented IC_50_ values ranging from 506 μM to 89 μM, and the main interactions were with Gln281 and His353,^32^ which are in accordance with the IC_50_ values described in this work. ACE-inhibitory peptides may even present other biological effects to improve blood pressure, such as those isolated from the collagens of monkfish (*Lophius litulon*) swim bladders, which increased nitric oxide production and decreased endothelin-1 levels.^12^ Peptides from Antarctic krill (*Euphausia superba*) hydrolysate also improved endothelial function by reducing oxidative damage.^33^ However, more potent ACE-inhibitory peptides are represented by peptides with lower IC_50_ values. Examples are the natural peptides VPP and IPP, obtained from sour milk, with IC_50_ values of 5 and 9 μM.^34^ Nanomolar range is expected in a potent ACE-inhibitory peptide, even though it is not easy to develop a nanomolar inhibitory molecule.

Aligning all characteristics of a potent ACE-inhibitory peptide described above, structural changes may be proposed to increase the inhibitory activity. Shortening of the sequence and the addition of hydrophobic and bulky groups are some suggestions that may be considered. Sequence optimization may also be applied to improve the peptide stability and protect the peptides from peptidases. As a perspective to evaluate the stability of the peptides, Caco-2 cells may be applied.^35^ In addition, its cytotoxicity potential should also be evaluated. The peptides presented in this study can serve as models for the design of novel and more potent peptides through sequence optimization, aiming for the development of a new ACE inhibitory and C-domain selective compound.

In summary, even though hypertension is considered the major cause of premature death worldwide and accounts for more deaths than cancers, it remains poorly controlled despite all available treatments. Although the industry has not applied much effort to develop more effective antihypertensive molecules, researchers have been studying the therapeutic application of bioactive peptides, as these molecules present several biological activities and great biocompatibility. In this study, we presented two bioactive peptides that present potential antihypertensive activity, evaluated through ACE inhibition. The peptides, PepDB_AHP1 and PepDB_AHP4, presented IC_50_ values of 331.2 and 88.6 μM, respectively. The difference between the two values may be explained due to the lack of hydrogen bonds between PepDB_AHP1 and ACE, as only 5 in 22 interactions are of this kind, while PepDB_AHP4 presents 12 hydrogen bonds out of 16 interactions. Although promising, it is important to notice that the inhibitory activity of the peptides may be improved using peptide optimization techniques, such as insertion of functional groups and noncanonical amino acids, shortening of the sequence, and cyclization. Also, its cytotoxicity should be evaluated in order to confirm the biocompatibility of the peptides and original and optimized sequences.

## Supplementary Material



## Notes

Full experimental details for the selection of the peptides, molecular docking, compounds synthesized and enzymatic assays (PDF)

## List of Abbreviations

ACE: angiotensin-converting enzyme
RAS: renin-angiotensin system
ACEis: angiotensin-converting enzyme inhibitors
AGT: angiotensinogen
Ang I: angiotensin I
Ang II: angiotensin II
tACE: testicular ACE
sACE: somatic ACE

## References

[1] World Health Organization. Hypertension. Available via the Internet at: https://www.who.int/news-room/fact-sheets/detail/hypertension. Accessed June 20, 2024.

[2] Nilson E. A. F., da Costa Santin Andrade R., de Brito D. A., de Oliveira M. L. (2020). Costs attributable
to obesity, hypertension, and diabetes in the Unified Health System,
Brazil, 2018. Rev. Panam. Salud Publica.

[3] Abdelkader N. N., Awaisu A., Elewa H., El Hajj M. S. (2023). Prescribing patterns
of antihypertensive medications: A systematic review of literature
between 2010 and 2020. Explor Res. Clin. Soc.
Pharm..

[4] Patel S., Rauf A., Khan H., Abu-Izneid T. (2017). Renin-angiotensin-aldosterone
(RAAS): The ubiquitous system for homeostasis and pathologies. Biomed. Pharmacother..

[5] Sturrock E. D., Natesh R., van Rooyen J. M., Acharya K. R. (2004). What’s new
in the renin-angiotensin system?. Cell. Mol.
Life Sci..

[6] Overlack A. (1996). ACE inhibitor-induced
cough and bronchospasm. Incidence, mechanisms and management. Drug Saf..

[7] Pearman N. A., Morris G. A., Smith A. M. (2025). Angiotensin-converting
enzyme (ACE)-inhibitor
activity of novel peptides derived from porcine liver and placenta. Molecules.

[8] de
Matos F. M., de Castro R. J. S. (2025). Characterization and identification
of potential antioxidant, antidiabetic, and antihypertensive peptides
from hydrolysates of Tenebrio molitor flour and its protein concentrate. J. Food Sci..

[9] Chen L., Cheng F., Chen H., Shu G. (2024). Preparation and identification
of novel angiotensin-I-converting enzyme inhibitory peptides from
Moringa oleifera leaf. Lebenson. Wiss. Technol..

[10] Cao X., Zhou H., Xie J., Zhang Z., Guo S., Luo J., Chen Q., Meng C., Zhang F., Hong J. (2025). A novel
angiotensin I-converting enzyme (ACE) inhibitory peptide
from Wuyi rock tea residue: Preparation, identification, and its potential
molecular mechanism. Lebenson. Wiss. Technol..

[11] Lin L., Yuan W., Xiao J., Jia J., Xie Y., Cai Q., Dai C., Li Q., Wang B. (2025). Identification
of novel ACE inhibitory peptides from Nannochloropsis oculata through
peptidomics, in silico screening and molecular docking. Food Chem..

[12] Hu Y.-D., Xi Q.-H., Kong J., Zhao Y.-Q., Chi C.-F., Wang B. (2023). Angiotensin-I-converting
enzyme (ACE)-inhibitory peptides from the
collagens of monkfish (*Lophius litulon*) swim bladders: Isolation, characterization, molecular docking analysis
and activity evaluation. Mar. Drugs.

[13] Suo S.-K., Zheng S.-L., Chi C.-F., Luo H.-Y., Wang B. (2022). Novel angiotensin-converting
enzyme inhibitory peptides from tuna byproducts-milts: Preparation,
characterization, molecular docking study, and antioxidant function
on H_2_O_2_-damaged human umbilical vein endothelial
cells. Front. Nutr..

[14] Xiang L., Zheng Z., Guo X., Bai R., Zhao R., Chen H., Qiu Z., Qiao X. (2024). Two novel
angiotensin I-converting enzyme inhibitory peptides from garlic protein:
In silico screening, stability, antihypertensive effects in vivo and
underlying mechanisms. Food Chem..

[15] Liu, Y. ; Zhang, L. ; Guo, M. ; Wu, H. ; Xie, J. ; Wei, D. Virtual screening for angiotensin I-converting enzyme inhibitory peptides from *Phascolosoma esculenta* . Bioresour. Bioprocess. 2014, 1 (1), 10.1186/s40643-014-0017-5.

[16] Pinzi L., Rastelli G. (2019). Molecular docking: Shifting paradigms in drug discovery. Int. J. Mol. Sci..

[17] Innocente-Alves, C. Identification of a synthetic peptide with potential angiotensin I-converting enzyme (ACE-1) inhibitory activity. Int. J. Pept. Res. Ther., 2025, 31 (2), 10.1007/s10989-024-10683-x.

[18] Maurer M. (2011). New topics in bradykinin
research. Allergy.

[19] Wei L., Alhenc-Gelas F., Corvol P., Clauser E. (1991). The two homologous
domains of human angiotensin I-converting enzyme are both catalytically
active. J. Biol. Chem..

[20] Natesh R., Schwager S. L. U., Evans H. R., Sturrock E. D., Acharya K. R. (2004). Structural
details on the binding of antihypertensive drugs captopril and enalaprilat
to human testicular angiotensin I-converting enzyme. Biochemistry.

[21] Fuchs S. (2008). Angiotensin-converting
enzyme C-terminal catalytic domain is the
main site of angiotensin I cleavage in vivo. Hypertension.

[22] Andújar-Sánchez M., Cámara-Artigas A., Jara-Pérez V. (2004). A calorimetric
study of the binding of lisinopril, enalaprilat and captopril to angiotensin-converting
enzyme. Biophys. Chem..

[23] Corradi H. R. (2007). The structure of testis
angiotensin-converting enzyme in complex
with the C domain-specific inhibitor RXPA380. Biochemistry.

[24] Watermeyer J. M., Kröger W. L., O’Neill H. G., Sewell B. T., Sturrock E. D. (2008). Probing
the basis of domain-dependent inhibition using novel ketone inhibitors
of Angiotensin-converting enzyme. Biochemistry.

[25] Danilov S.
M., Balyasnikova I. V., Albrecht R. F., Kost O. A. (2008). Simultaneous determination
of ACE activity with 2 substrates provides information on the status
of somatic ACE and allows detection of inhibitors in human blood. J. Cardiovasc. Pharmacol..

[26] Cheung H. S., Wang F. L., Ondetti M. A., Sabo E. F., Cushman D. W. (1980). Binding
of peptide substrates and inhibitors of angiotensin-converting enzyme.
Importance of the COOH-terminal dipeptide sequence. J. Biol. Chem..

[27] Shirley B. A., Stanssens P., Hahn U., Pace C. N. (1992). Contribution of
hydrogen bonding to the conformational stability of ribonuclease T1. Biochemistry.

[28] Pace C. N., Shirley B. A., McNutt M., Gajiwala K. (1996). Forces contributing
to the conformational stability of proteins. FASEB J..

[29] Fu Y., Alashi A. M., Young J. F., Therkildsen M., Aluko R. E. (2017). Enzyme inhibition kinetics and molecular interactions
of patatin peptides with angiotensin I-converting enzyme and renin. Int. J. Biol. Macromol..

[30] Ye S. (2022). Isolation and identification
of novel angiotensin I-converting enzyme
(ACE) inhibitory peptides from Pony Seed and evaluation of the inhibitory
mechanisms. J. Funct. Foods.

[31] Lu Y. (2021). Inhibitory mechanism
of angiotensin-converting enzyme inhibitory
peptides from black tea. J. Zhejiang Univ. Sci.
B.

[32] Tang H., Wang C., Cao S., Wang F. (2022). Novel angiotensin I-converting
enzyme (ACE) inhibitory peptides from walnut protein isolate: Separation,
identification and molecular docking study. J. Food Biochem..

[33] Zhu W.-Y., Wang Y.-M., Dong X.-M., Zhao G.-X., Chi C.-F., Wang B. (2025). Antioxidant peptides from Antarctic krill (Euphausia superba) hydrolysate:
Stability, ACE inhibitory activity, and endothelial cells protection
by regulating Keap1/Nrf2 pathway. J. Agric.
Food Res..

[34] Nakamura Y., Yamamoto N., Sakai K., Okubo A., Yamazaki S., Takano T. (1995). Purification and characterization of angiotensin I-converting
enzyme inhibitors from sour milk. J. Dairy Sci..

[35] Sangsawad P. (2018). Transepithelial transport across Caco-2 cell monolayers of angiotensin
converting enzyme (ACE) inhibitory peptides derived from simulated
in vitro gastrointestinal digestion of cooked chicken muscles. Food Chem..

