# Supplementary material for: Characterization of Novel Angiotensin-Converting Enzyme Inhibitory Peptides
Source: ACS Med Chem Lett. 2025 Nov 26;16(12):2486–91. doi: 10.1021/acsmedchemlett.5c00569 (PMC12703502; doi:10.1021/acsmedchemlett.5c00569)
Supplement: Supplementary file 1 [file ml5c00569_si_001.pdf]

Characterization of Novel Angiotensin-Converting Enzyme Inhibitory Peptides

Camila Innocente-Alves[1,2], Sara Luísa Sulzbach[1], Emerson Gonçalves Moreira[3], Raul Izidoro Carneiro[3], Lucélia Santi[1,2,3], Hugo Verli[2,3], Walter Orlando Beys-da-Silva[1,2,3*]

[1] Faculdade de Farmácia, Universidade Federal do Rio Grande do Sul, Porto Alegre, Brasil

[2] Programa de Pós-Graduação em Biologia Celular e Molecular, Universidade Federal do Rio Grande do Sul, Porto Alegre, Brasil

[3] Centro de Biotecnologia (CBiot), Universidade Federal do Rio Grande do Sul, Porto Alegre, Brasil

[*] corresponding author: walter.beys@ufrgs.br

Experimental procedures

Selection of the bioactive peptides

The peptides were selected from a previous virtual screening performed against our chemical library (PepDB) [1], and evaluated *in vitro* for its antihypertensive potential. The structures of the peptides, MSFLEHFLELK (PepDB_AHP1, molecular mass 1393.66 g/mol) and VWTNCYHLYPAH (PepDB_AHP4, molecular mass 1503.70 g/mol), were built using PyRosetta [2]. The enzyme structure was obtained from the Protein Data Bank (PDB), reference 1UZE, and prepared using Maestro Protein Prep Wizard software [3] at 8.3 pH. The peptides, enzyme, and Zn^{2+} cofactor structures were uploaded to the DockThor server [4] for molecular docking. The grid parameters used were set as follows: the grid center coordinates were X = 42, Y = 35, and Z = 43, with a grid size of X = 31, Y = 37, and Z = 31, and a discretization of 0.34. The molecular docking result was evaluated using the Protein-Ligand Interaction Profile (PLIP) tool (https://plip-tool.biotec.tu-dresden.de/) for characterization of the interactions and PyMOL (https://www.pymol.org/) visualization.

Angiotensin-converting enzyme inhibitory assays

The peptides were synthesized by AminoTech (São Paulo, Brazil). All compounds are >95% pure by HPLC. The ability of the peptides to inhibit ACE activity was evaluated by the Cushman & Cheung method [5] with slight modifications [1]. Absorbances were read at 228 nm (Spectramax M2, Molecular Devices) and ACE inhibition was expressed as percentage of inhibition. The percentage of inhibition was calculated using the following formula:

$$\% \text{ Inhibitory Activity} = [\,1 - (A - C) / (B - D)]\times 100$$

Where:

A = HHL + potential inhibitor + ACE

B = HHL + ACE

C = HHL + potential inhibitor

D = HHL

Statistical analysis

All the experiments were performed in triplicates, at least. The IC50 values, defined as the concentration of the peptides required to inhibit 50% of the enzyme's activity, were calculated by nonlinear regression using GraphPad Prism 8.0.1 (GraphPad Software Inc., San Diego, California, USA).

References

[1] C. Innocente-Alves et al., “Identification of a synthetic peptide with potential angiotensin I-converting enzyme (ACE-1) inhibitory activity,” Int. J. Pept. Res. Ther., vol. 31, no. 2, Jan. 2025, doi: 10.1007/s10989-024-10683-x.

[2] S. Chaudhury, S. Lyskov, and J. J. Gray, “PyRosetta: a script-based interface for implementing molecular modeling algorithms using Rosetta,” Bioinformatics, vol. 26, no. 5, pp. 689–691, Mar. 2010, doi: 10.1093/bioinformatics/btq007.

[3] G. M. Sastry, M. Adzhigirey, T. Day, R. Annabhimoju, and W. Sherman, “Protein and ligand preparation: parameters, protocols, and influence on virtual screening enrichments,” J. Comput. Aided Mol. Des., vol. 27, no. 3, pp. 221–234, Mar. 2013, doi: 10.1007/s10822-013-9644-8.

[4] I. A. Guedes et al., “DockThor-VS: A Free Platform for Receptor-Ligand Virtual Screening,” J. Mol. Biol., p. 168548, Mar. 2024, doi: 10.1016/j.jmb.2024.168548.

[5] D. W. Cushman and H. S. Cheung, “Spectrophotometric assay and properties of the angiotensin-converting enzyme of rabbit lung,” Biochem. Pharmacol., vol. 20, no. 7, pp. 1637–1648, Jul. 1971, doi: 10.1016/0006-2952(71)90292-9.
